# Molecular viability testing of viable but non‐culturable bacteria induced by antibiotic exposure

**DOI:** 10.1111/1751-7915.13039

**Published:** 2017-12-15

**Authors:** Seunguk Lee, Sungwoo Bae

**Affiliations:** ^1^ Department of Civil and Environmental Engineering National University of Singapore 1 Engineering Drive 2 Singapore 117576 Singapore

## Abstract

Nucleic acid amplification‐based methods are limited by their inability to discriminate between viable and dead cells. To overcome this drawback, propidium monoazide (PMA) combined with qPCR has been used to differentiate viable from nonviable cells in environmental samples. However, assessing bacterial physiology using PMA‐qPCR remains a challenge due to its incapability of detecting metabolic activities, leading to overestimation of the viable bacteria population under an inactivation condition (e.g. antibiotic treatments). A recent advanced technique to amplify ribosomal RNA precursors (pre‐rRNA) has been shown to detect viable cells because pre‐rRNAs are intermediates in rRNA synthesis. This study investigated the effect of different types of antibiotics on the bacterial viability or viable but non‐culturable (VBNC) state using both PMA‐qPCR and pre‐rRNA analyses with *Pseudomonas aeruginosa*. This study demonstrated that *P. aeruginosa* was more sensitive to colistin than it was to carbenicillin, gentamicin and levofloxacin. We could discriminate VBNC *P. aeruginosa* cells using PMA‐qPCR when antibiotic pressure induced the VBNC state. Also, pre‐rRNA was able to distinguish viable cells from colistin‐inactivated bacteria cells, and it could detect the presence of VBNC and persister cells. Our results showed that these two molecular methods could successfully eliminate false‐positive signals derived from antibiotics‐inactivated cells.

## Introduction

Cultivation is one of the most fundamental steps in enumerating viable bacteria for food‐ and water‐quality assessment. However, the conventional culture methods used to quantify specific microorganisms are time‐consuming and unreliable, especially when bacteria enter an unculturable life stage in response to harsh environments. Many bacterial species enter a transient state of ‘dormancy’ as a strategy to survive unfavourable conditions (Lennon and Jones, 2011). In this state, the bacteria cannot be detected using culture‐based methods, but they remain viable and retain virulence under environmental stressors. Two well‐defined dormancy states, the viable but non‐culturable (VBNC) state and persister cells (persisters), have been described in non‐sporulating bacteria, where they often exhibit strong antibiotic tolerances than culturable cells (viable) (Li *et al*., 2014; Maisonneuve and Gerdes, 2014). In the VBNC state, bacteria maintain a low metabolic activity and do not divide, which help them cope with chemical stressors such as chlorination (Oliver *et al*., 2005) and antibiotic treatments (Ayrapetyan *et al*., 2015a). Even so, they could resuscitate and regrow later when the environmental conditions are favourable. In the early 1940s, Bigger first introduced the term ‘persister cells’ to mean cells that are genetically identical to antibiotic susceptible bacteria, but phenotypically different as they are tolerant to antimicrobial agents (Bigger, 1944; Wiuff *et al*., 2005; Lewis, 2010). Persister cells exhibit antibiotic tolerances similar to VBNC cells (Ayrapetyan *et al*., 2015b). Thus, these two modes (VBNC state and persister cell) produce antibiotic‐tolerant bacteria populations and pose a public health risk because of regaining virulence after antibiotic treatment (Nowakowska and Oliver, 2013; Helaine and Kugelberg, 2014).

Alternatively, rapid and versatile nucleic acid‐based techniques that detect specific DNA and RNA genes have been used to identify and quantify bacteria in environmental samples (Malorny *et al*., 2003; Yanez *et al*., 2011). Despite the advantages of culture‐independent methods, the limitation of molecular assessment (especially for DNA‐based methods) is the possible overestimation of viable cell densities because DNA can persist for an extended period after cell death in environments (Rudi *et al*., 2005). Recently, DNA‐intercalating dyes such as ethidium monoazide (EMA) and propidium monoazide (PMA) have been proposed as useful molecular methods to quantify intact bacterial cells in environments (Nogva *et al*., 2003; Rudi *et al*., 2005; Nocker *et al*., 2006). PMA or EMA with PCR technique has been widely used to assess cell viability in water, soil, air and food (Rogers *et al*., 2008; Bae and Wuertz, 2009; Miotto *et al*., 2012; Kaushik and Balasubramanian, 2013; Li and Chen, 2013).

A drawback of DNA‐intercalating dyes is that they are ineffective in discriminating metabolically active cells due to the viability criterion of membrane integrity (Nocker and Camper, 2009). To overcome this limitation, recent studies have proposed detecting microbial rRNA precursors (pre‐rRNA) as an alternative to rapidly detecting viable cells (Cangelosi *et al*., 2010; Weigel *et al*., 2013, 2017; Cangelosi and Meschke, 2014; Do *et al*., 2014; Spooner *et al*., 2016). Pre‐rRNA fractions in growing bacteria cells are significant in the total RNA, and they are much easier to detect than mRNAs as they synthesize rRNA precursors (pre‐rRNA) immediately in response to the nutritional stimulation of bacterial cells (Cangelosi *et al*., 2010). The method of quantifying pre‐rRNA precursors has been evaluated for differentiating viable cells from dead cells that have been inactivated by chlorine, serum exposure, pasteurization and UV irradiation (Cangelosi *et al*., 2010; Weigel *et al*., 2013, 2017; Do *et al*., 2014).

Thus, both the pre‐rRNA or PMA‐qPCR assays could be alternative methods for quantifying VBNC cells. However, it is still uncertain whether pre‐rRNA analysis or PMA‐qPCR will enable researchers to differentiate VBNC from dead cells during antimicrobial treatment because bacterial cells could enter the VBNC state, producing an antimicrobial‐tolerant population capable of withstanding prolonged lethal treatment (Ayrapetyan *et al*., 2015a). The objective of this study therefore was to employ PMA‐qPCR and pre‐rRNA analyses to assess the bacterial viability and VBNC state during antimicrobial treatment. We chose *Pseudomonas aeruginosa* as a model bacterium because the well‐recognized opportunistic pathogen can enter a VBNC state and be resuscitated later (Silby *et al*., 2011; Zhang *et al*., 2015). To the best of our knowledge, no previous study has evaluated PMA‐qPCR and pre‐rRNA analyses as a method to distinguish VBNC from dead cells inactivated by antibiotics.

## Results

### Optimization of the PMA‐qPCR for *P. aeruginosa* PAO1

**Figure 1:**
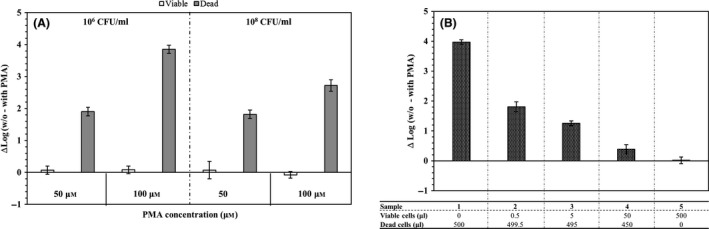
Optimization of the PMA‐PCR assay for wild‐type *Pseudomonas aeruginosa* PAO1. Results are shown as differences in ΔLog (w/o – with PMA) values by subtracting the log copies values of PMA‐treated from the log copies values of PMA‐untreated samples. (A) The white bars and grey bars show viable and dead cells respectively. (B) The table shows mixing ratios of viable/dead cells. The error bars represent means and standard deviations, which were obtained in three independent replicates.

The effects of the PMA treatment under different cell densities were determined using qPCR assays targeting between the 5′ terminus of the mature 16S rRNA and the external transcribed spacer (ETS1) derived from viable and heat‐killed cells, as described previously (Cangelosi *et al*., 2010; Weigel *et al*., 2013; Do *et al*., 2014). For a 100 μM concentration, there was a 2.7 to 3.9 log reduction, and there was a 1.8 to 1.9 reduction in a 50 μM concentration of PMA (Fig. 1A). The log reduction values at a 100 μM concentration of PMA for the dead cells were higher than those at a 50 μM concentration. In this study, the concentration of 100 μM PMA and cell density at 10^6^ CFU ml^−1^ were the best at distinguishing viable cells from heat‐killed cells. Thus, the concentrations of PMA and the bacterial suspensions were chosen in our study.

Next, we evaluated the ability of PMA to differentiate viable cells from dead cells in the mixture of viable and dead *P. aeruginosa* PAO1, as shown in Fig. 1B. The number of gene copies without PMA showed no differences within a mixture of viable and dead cells from wild‐type PAO1 (*P *> 0.05), whereas the number of gene copies with PMA increased gradually with increasing proportions of viable cells resulting in ΔLog_10_ values decreasing (Fig. 1B). These results clearly showed that the PMA‐qPCR could effectively quantify viable cells in the mixture of viable and heat‐killed *P. aeruginosa* PAO1 cells.

### Monitoring of antibiotics treatment in *P. aeruginosa* PAO1 by PMA‐qPCR

**Figure 2:**
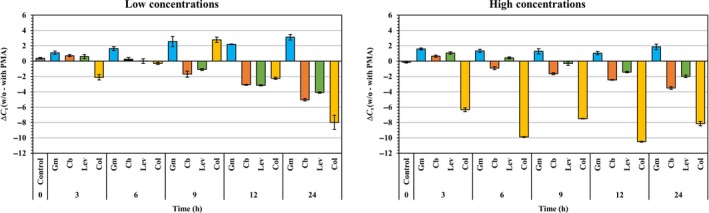
Effect on different concentrations of antibiotics by PMA‐qPCR. Results are shown by ΔC_t_ values after 24 h of incubation with four antibiotics (gentamicin, carbenicillin, levofloxacin and colistin). These values were calculated by subtracting the average *C*_*t*_ values of PMA‐treated from the average *C*_*t*_ values of PMA‐untreated samples. The left result shows the low concentrations of antibiotics (Gm = 12, CB = 300, lev = 3 and col = 6 μg ml^−1^), and the right result shows high concentrations of antibiotics (Gm = 24, CB = 600, lev = 6 and col = 12 μg ml^−1^). The error bars represent means and standard deviations, which were obtained in three independent replicates. Gm, gentamicin; Cb, carbenicillin; Lev, levofloxacin; Col, colistin.

To test for the effect of antibiotic use on cell viability, the viable *P. aeruginosa* PAO1 cells were enumerated using PMA‐qPCR under low and high concentrations of the antibiotics, as shown in Fig. 2. The Δ*C*_*t*_ values (w/o – with PMA) showed that the types of antibiotics and their concentrations affected the cell viability (Fig. 2). The percentage of VBNC cells were also evaluated in comparison with total cells. When *P. aeruginosa* PAO1 cells were treated with 12 or 24 μg ml^−1^ gentamicin (Gm, blue colours), the cell viability from the PMA‐qPCR assay exhibited no differences in ΔC_t_ regardless of exposure times (Fig. 2), and the percentage of VBNC cells were 88% to 92% under low concentrations and 95% to 98% under high concentrations. After the 9 h exposure, however, the carbenicillin‐treated and levofloxacin (Cb, orange colours; Lev, blue colours)‐treated cells started to decrease in Δ*C*_*t*_ (Fig. 2) and the percentage of VBNC cells (data not shown), indicating that a longer exposure time to the antibiotics inactivated the cells. The Δ*C*_*t*_ values of *P. aeruginosa* PAO1 cells treated with 6 or 12 μg ml^−1^ colistin (Col, yellow colours) decreased from 2.1 to 10.5 after being incubated for 24 h. Unlike other antibiotics, the percentage of VBNC cells treated with colistin fell from 82% to 56% under low concentrations and from 65% to 59% under high concentrations, although slight fluctuations were observed during the 24 h of incubation.

### Pre‐ribosomal RNA precursor (pre‐rRNA) analysis for *P. aeruginosa* PAO1 with antibiotics exposure

**Figure 3:**
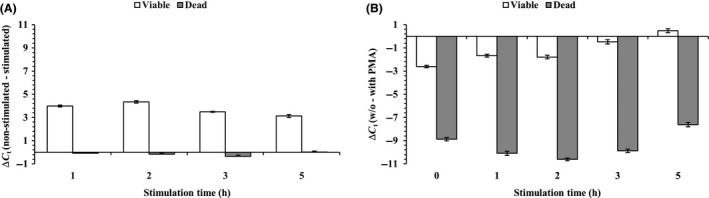
Pre‐rRNA and PMA‐qPCR analyses in viable and heat‐killed cells. (A) The Δ*C*_*t*_ values are calculated by subtracting nutritionally stimulated samples from the non‐stimulated samples based on RT‐qPCR. (B.) The white and grey bars show Δ*C*_*t*_ values that were calculated by subtracting the average *C*_*t*_ values of PMA‐treated from the average *C*_*t*_ values of PMA‐untreated samples. The error bars represent means and standard deviations, which were obtained in three independent replicates.

To validate whether pre‐rRNA synthesis could distinguish viable cells from dead cells by brief nutritional stimulation, time‐courses of nutritional stimulation were applied for viable and heat‐killed *P. aeruginosa* PAO1 cells. The PMA‐qPCR was also employed to detect viable cells and heat‐killed bacteria in the same samples after nutritional stimulation that had been used in the parallel pre‐rRNA experiment. The Δ*C*_*t*_ values for viable *P. aeruginosa* PAO1 cells showed positive values in the range of Δ*C*_*t*_ between 3.1 and 4.3 (Fig. 3A, white bars), indicating that only viable cells were nutritionally stimulated by the addition of a fresh culture medium. The highest Δ*C*_*t*_ values for viable cells were reached with a nutritional stimulation time of 2 h. The pre‐rRNA was very rapidly synthesized by one to two hours of nutritional stimulation in *P. aeruginosa* PAO1. However, heat‐killed *P. aeruginosa* PAO1 cells did not synthesize pre‐rRNA upon nutritional stimulation (Fig. 3A, grey bars). It is worth noting that the Δ*C*_*t*_ values with or without a PMA treatment on viable cells did not show dramatic changes (white bars; *P *> 0.05), whereas a considerable difference in Δ*C*_*t*_ values (grey bars; *P *< 0.05) was observed for dead cells regardless of the nutritional stimulation times (Fig. 3B).

### Application of the pre‐rRNA and PMA‐qPCR assay to VBNC/persister *P. aeruginosa* PAO1 cells induced by different antibiotics

**Figure 4:**
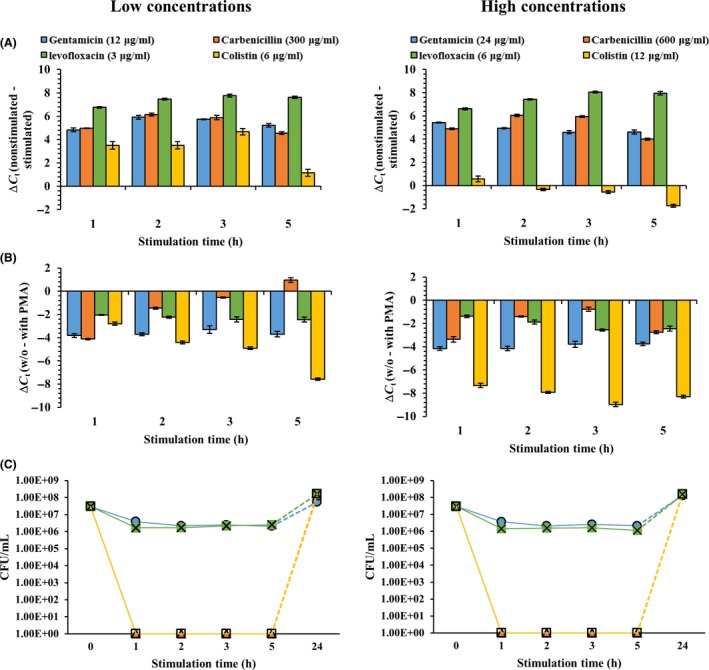
Pre‐rRNA, PMA and colony‐counting analysis in *Pseudomonas aeruginosa* PAO1 after exposure to antibiotics based on RT‐qPCR, PMA‐qPCR and CFU ml^−1^. (A) The Δ*C*_*t*_ values are calculated by subtracting nutritionally stimulated samples from the non‐stimulated samples based on RT‐qPCR. (B) The Δ*C*_*t*_ values were calculated by subtracting the average *C*_*t*_ values of PMA‐treated from the average *C*_*t*_ values of PMA‐untreated samples. (C) The CFU ml^−1^ results are shown. Dotted lines represent the resuscitation of *P. aeruginosa* cells from the VBNC or persister cells after antibiotics are removed. The left result shows low concentrations of antibiotics, and the right result shows high concentrations of antibiotics. The error bars represent means and standard deviations, which were obtained in three independent replicates.

The pre‐rRNA and PMA‐qPCR were applied to understand the VBNC state present when exposed to different types of antibiotics, while plate counting was conducted to check the cultivability of *P. aeruginosa* PAO1. When *P. aeruginosa* PAO1 cells were treated with 3 or 6 μg ml^−1^ of levofloxacin (lev, green colours), the result of the pre‐rRNA assay showed an increase in Δ*C*_*t*_ values from 6.6 to 8.1. The Δ*C*_*t*_ values increases were probably due to the ineffective inactivation by levofloxacin (Fig. 4A). Similarly, the Δ*C*_*t*_ values of gentamicin (Gm, blue colours)‐treated and carbenicillin (Cb, orange colours)‐treated *P. aeruginosa* PAO1 cells also increased within a 5 h stimulation (Fig. 4A). The Δ*C*_*t*_ values of *P. aeruginosa* PAO1 cells that were treated with a concentration of colistin (Col, yellow colours) also increased within a 5 h stimulation, but the difference was less than what was seen with other antibiotics (Fig. 4A). Likewise, the Δ*C*_*t*_ values for colistin obtained from the PMA‐qPCR assay were similarly decreased in agreement with the pre‐rRNA results (Fig. 4B). In high‐dose antibiotics, the colistin treatment caused negative Δ*C*_*t*_ values as compared to those treated with other antibiotics. Also, the Δ*C*_*t*_ values of the colistin treatment from PMA‐qPCR showed significant differences as compared to those under different antibiotics.

Furthermore, the number of *P. aeruginosa* PAO1 cells (CFU) decreased for the first hour only with levofloxacin (3 or 6 μg ml^−1^) and gentamicin (12 or 24 μg ml^−1^), but subsequently they resumed *growing* but maintained the same cell concentration (approximately 10^6^ CFU ml^−1^) during the 5 h of incubation (Fig. 4C). However, the CFU of culturable *P. aeruginosa* PAO1 were undetectable when only treated with colistin and carbenicillin (Fig. 4C) while viable *P. aeruginosa* PAO1 were still detectable with qPCR even though the wild‐type cells were treated with the antibiotics. Resuscitation from antibiotics‐inactivated bacteria cells was also performed, resulting in approximately 10^8^ CFU ml^−1^ after a 24 h resuscitation period (Fig. 4C, dotted lines). These results demonstrated that VBNC and persister cells induced by an antibiotics treatment could resuscitate and subsequently regrow when the antibiotics were removed.

## Discussion

The results of this study showed that cell viabilities of *P. aeruginosa* PAO1 measured by PMA‐qPCR were more sensitive to colistin than to the other antibiotics. Recently, PMA‐qPCR was used to identify intact cells from antibiotic‐inactivated *Staphylococcus aureus* and *S. epidermidis* with vancomycin and gentamicin (Kobayashi *et al*., 2010). This study reported that the *C*_*t*_ difference (*Ct*_with PMA_ – *Ct*_without PMA_) under gentamicin treatments increased gradually, but decreased after a 24 h of incubation, indicating that the number of viable bacteria had increased and was still present after a 24 h of incubation. Similarly, our PMA‐qPCR study showed wild‐type *P. aeruginosa* PAO1 cells still remained intact under the gentamicin treatment. An interesting finding was that most of the cells exposed to antibiotics (carbenicillin, colistin, gentamicin and levofloxacin) were not detectable on the culture‐based methods (data not shown). We concluded that the antibiotic pressure of gentamicin could induce the viable but non‐culturable state (VBNC) of *P. aeruginosa* PAO1 because the Δ*C*_*t*_ values were positive and unchanged over the 24 h of incubation. In contrast, the other three antibiotics (carbenicillin, levofloxacin and colistin) inactivated *P. aeruginosa* PAO1 cells after the 24 h of incubation. A possible explanation for the above result is that this was caused partly by different mechanisms of action in each antibiotic. Gentamicin is an aminoglycoside which causes protein synthesis inhibition by targeting to the 30S ribosomal (Neu, 1992; McManus, 1997), but levofloxacin is a quinolone antibiotic which acts by inhibiting the topoisomerase IV or DNA gyrase (Aldred *et al*., 2014). Carbenicillin is a semi‐synthetic antibiotic related to penicillin (belonging to β‐lactams) which interferes with cell wall synthesis in peptidoglycan cross‐linking, and colistin (also called polymyxin E) is a polypeptide antibiotic belonging to the polymyxin group which binds to lipopolysaccharide (LPS) and phospholipids in the membrane of Gram‐negative bacteria causing cell death (Davis *et al*., 1971; Evans *et al*., 1999). The antibiotic effect of carbenicillin and colistin therefore might have a much more direct effect on the cell wall and periplasm membrane as compared to gentamicin and levofloxacin. A recent study by Tavernier and Coenye showed that *P. aeruginosa* cells were more sensitive to colistin than levofloxacin in biofilm condition using PMA‐qPCR (Tavernier and Coenye, 2015). The interference of antibiotics with the cell wall and periplasm membrane synthesis might affect the PMA treatment because the DNA‐binding chemical is a membrane‐impermeant dye that selectively penetrates cells with compromised membranes, which can be considered dead cells (Nocker *et al*., 2006). A previous study also showed that vancomycin (inhibition of cell wall synthesis) was more efficient at inhibiting PCR amplification on PMA treatment than gentamicin (Kobayashi *et al*., 2010). Thus, PMA‐qPCR could be used to discriminate VBNC from antibiotics‐inactivated bacteria cells when antibiotic pressure induces the VBNC state in *P. aeruginosa* PAO1, which is known to be an opportunistic pathogen‐ and antibiotic‐resistant bacterium. However, PMA‐qPCR based on cell membrane integrity might be incapable of discriminating VBNC cells from other conditions such as UV irradiation (Leifels *et al*., 2015; Weigel *et al*., 2017) because the cell membrane of the VBNC state might be still intact meaning the cell will be identified as live. This will result in an overestimation of VBNC cells. As an alternative to this viability method, pre‐rRNA has been successfully applied to discriminate viable cells from chlorine‐, serum‐, UV‐ and low‐temperature pasteurization (63 °C for 45 min)‐killed cells of *A. hydrophila*,* P. aeruginosa, L. monocytogenes*,* M. avium*,* E. coli*,* E. faecalis* and *S. enterica* (Cangelosi *et al*., 2010; Weigel *et al*., 2013, 2017; Do *et al*., 2014). Despite the successful application of the pre‐rRNA analysis, its efficacy for VBNC detection has remained problematic under environmental stressors. Based on pre‐rRNA results, the pre‐rRNA method can eliminates false‐positive signals derived from colistin‐inactivated cells, but not gentamicin, carbenicillin and levofloxacin after nutritional stimulation. It is possible that carbenicillin, gentamicin and levofloxacin could not completely inactivate *P. aeruginosa* PAO1 cells, as in the PMA experiment above. Also, the step of having a 7 day starvation before the stimulation could induce VBNC cells because they have intact cell membranes, low metabolic activity and continued mRNA synthesis (Lleo *et al*., 2000). It has been known that many species of bacteria enter VBNC state when they are subjected to various environmental stressors, such as UV disinfection, osmotic pressures, high/low temperature and starvation (Colwell *et al*., 1985; Byrd *et al*., 1991; Oliver *et al*., 2005; Pawlowski *et al*., 2011). Interestingly, it has been demonstrated that VBNC and persister cells coexist after antibiotic treatment and can be resuscitated back to culturable cells when the existing stress is removed (Ayrapetyan *et al*., 2015b). Thus, *P. aeruginosa* PAO1 cells were induced into a VBNC or persistence state when treated with four different types of antibiotics (carbenicillin, colistin, gentamicin and levofloxacin).

In summary, our study revealed that a starvation step prior to nutrient stimulation could induce VBNC or persister cells, as shown in Figs 3 and 4. In addition, the suitability of using pre‐rRNA to detect VBNC cells is further validated under environmental stressors. To the best of our knowledge, this is the first study to demonstrate that two molecular viability tests (pre‐rRNA and PMA‐qPCR) were able to discriminate viable cells from colistin‐inactivated bacteria cells and to detect the presence of VBNC and persister cells in a sample of *P. aeruginosa* PAO1. Overall, pre‐rRNA could be a promising technique for the detection of VBNC cells in comparison with PMA‐qPCR. These methods are dependent on the type of antibiotic, so further studies are needed to investigate the effect of many different types of antibiotics on bacterial viability in environments.

## Experimental procedures

### Bacterial strains and culture conditions

Wild‐type *P. aeruginosa* PAO1 strain was used as a model bacterium in this study. *P. aeruginosa* PAO1 strain was grown in Luria broth (LB) and LB agar (Sigma‐Aldrich, USA) in the presence or absence of different antibiotics at 37 °C. Each strain was grown in 5 ml of LB broth at 37 °C for 15 h at 200 rpm in a shaking incubator. The cells were adjusted at 0.05 for optical density at 600 nm (OD_600_) to be transferred into fresh LB medium, and then the medium was incubated in a rotating shaker under the same conditions as described above. Cell growth was monitored by measuring OD_600_ throughout the inoculation period. Colony‐forming units (CFU) were counted by spreading the serially diluted PBS in a 10‐fold suspension of bacteria on LB agar plates for a 24 h of incubation period at 37 °C. Antibiotics (carbenicillin, Colistin, gentamicin and levofloxacin) were added as needed to inhibit bacterial growth.

### PMA treatment

The PMA (Biotium, Hayward, CA, USA) was dissolved in ddH2O to make a stock concentration of 20 mM and stored at −20 °C in the dark before use. PMA was added to 500 μl aliquots of bacterial culture to a final concentration of either 50 or 100 μM, and then the cultures were incubated for 5 min in the dark at room temperature. Samples were then exposed to intense visible light using the PMA‐Lite™ LED Photolysis device (Biotium, Hayward, CA, USA) for 15 min. After the photo‐induced cross‐linking, the samples were pelleted at 5000 × *g* for 10 minutes and DNA was extracted from the pallet.

To evaluate the efficacy of the PMA, the concentration of various viable cells in suspensions in proportions of 0%, 0.1%, 1%, 10% and 100% was mixed with heat‐inactivated cells (maintaining an equal number of cells, 10^6^ CFU ml^−1^). Heat‐killed cell suspensions were prepared by being boiled at 99 °C for 10 min. After the heat treatment, the absence of viable cells was confirmed when the samples were cultured on a LB agar plate. Each viable and heat‐killed cell mixture was added to the PMA to a final concentration of 100 μM.

### PMA‐qPCR analysis

For analysis of antibiotics effect on PMA‐qPCR, wild‐type *P. aeruginosa* PAO1 cells were used. After growing for 3 h in a fresh medium, the cells were cultivated in 100 ml of LB broth containing 300 or 600 μg ml^−1^ carbenicillin, 6 or 12 μg ml^−1^ colistin, 12 or 24 μg ml^−1^ gentamicin and 3 or 6 μg ml^−1^ levofloxacin respectively. The antibiotic concentrations used in the experiments were threefold to sixfold minimal inhibitory concentrations (MICs) determined by the European Committee on Antimicrobial Susceptibility Testing (EUCAST) guidelines: http://www.eucast.org/zone_diameter_distributions. The cultures were incubated for 3, 6, 9, 12 and 24 h at 37 °C while being shaken at 200 rpm Each sample was also plated on LB agar plates to determine the CFU analysis. The results of the PMA‐qPCR experiments were expressed as a negative in Δ*C*_*t*_ by subtracting the *C*_*t*_ value of the PMA‐treated from the average *C*_*t*_ values of PMA‐untreated samples.

### Nutritional stimulation and Pre‐rRNA analysis

To assess the effects of pre‐rRNA replenishment over the period of nutritional stimulation, we followed procedures detailed in previous studies (Cangelosi and Meschke, 2014). Briefly, *P. aeruginosa* PAO1 strain was grown at 37 °C in 5 ml LB broth while being shaken at 200 rpm for 15 h, and then, it was transferred to 100 ml of LB broth in 250 ml flasks with its OD_600_ adjusted to be 0.05. It was then incubated for 15 h at 37 °C. The newly grown cells in 10 ml of the broth were centrifuged and washed two times with 1× phosphate‐buffered saline (PBS, 137 mM NaCl, 2.7 mM KCl and 10 mM Phosphate Buffer, pH 7.4) (Vivantis Technologies, Malaysia). After washing, the cell suspensions were re‐suspended in a 10 ml PBS buffer and transferred to 100 ml of LB broth. The *P. aeruginosa* PAO1 was incubated for 7 days to become nutrient‐limited bacterial cells. After 7 days of incubation, each sample was divided into two 500 μl aliquots; samples were inoculated into fresh LB broth (nutritional stimulation) and the other PBS buffer (non‐stimulated) respectively. During the nutritional stimulation or starvation period, samples were collected at time = 0 (non‐stimulated), 1, 2, 3 and 5 h for DNA and RNA, and PMA analysis respectively. At the same time, samples were plated on LB agar for CFU analysis. This procedure was repeated in triplicate. The results of the pre‐rRNA experiments were expressed as a positive in Δ*C*_*t*_ by subtracting the *C*_*t*_ value of the nutritionally stimulated samples from the non‐stimulated samples, as described in a previous study (Do *et al*., 2014). Based on Do *et al*., if there is a Δ*C*_*t*_ greater than one, it can be concluded that pre‐rRNA was synthesized (Do *et al*., 2014).

To analyse the effect of antibiotics on pre‐rRNA and PMA‐qPCR, we applied the same dosage of antibiotics as described above. Prior to nutritional stimulation, different antibiotics, as described above, were dosed into the 100 ml of LB broth in a 250‐ml flask. After 7 days of starvation (no additional nutrients), the bacterial suspensions were subsequently supplemented with a fresh medium containing antibiotic and then incubated at 37 °C for 5 h. Each sample was collected at 0, 1, 2, 3 and 5 h for pre‐rRNA and PMA‐qPCR analyses and plated on agar plates for counting colonies, according to the method described above. After the removal of the four different types of antibiotics, the VBNC cells’ resuscitation was also measured using spread plating. Samples were taken from antibiotics‐inactivated bacteria cells after five hours of incubation. The cells were pelleted by centrifugation at 5000 × *g* for 10 min, washed four times with 1× PBS buffer to remove the antibiotics, and then the cells were re‐suspended in 1 ml of PBS. The cell suspensions were then serially diluted in 10‐fold PBS and plated on LB agar plates at 37 °C for 24 h.

### DNA and RNA extraction

All bacterial DNA extractions were performed using a DNeasy Blood and Tissue Kit (Qiagen, Valencia, CA, USA) following the manufacturer's protocol. Three replicates were prepared for one experiment, and at least two independent experiments were performed for each experimental group.

RNA extractions from the *P. aeruginosa* PAO1 strain were performed; the bacterial cells were collected from the suspensions at each time point. We used the RNA Protect Bacterial Reagent (Qiagen, Valencia, CA, USA) to stabilize the total RNA in the bacterial cultures. RNA extraction subsequently was performed using the PureLink RNA Mini Kit (Ambion, Life Technologies, USA) according to the manufacturer's instructions. The eluted RNAs were treated with on‐column PureLink DNase (Ambion, Life Technologies, USA) prior to analysis, according to the manufacturer's protocol. cDNA was synthesized using a SuperScript IV (Invitrogen) enzyme from each RNA. A NanoDrop 2000 spectrophotometer (Thermo Fisher Scientific, Wilmington, DE, USA) was used to quantify the DNA and RNA concentrations.

### cDNA synthesis and real‐time PCR assays

Quantitative PCR (qPCR) assays were performed to amplify genomic DNA (gDNA) and cDNA in a MicroAmp optical 96‐well reaction plate using an automated ABI Step‐One‐Plus Real‐Time PCR system (Applied Biosystems, CA, USA). For pre‐RNA quantification, cDNA was synthesized from each RNA using the primers in the previous report (Weigel *et al*., 2013). Reverse and cDNA synthesis primers recognized sequences within the mature rRNA (16S) while forward primers recognized species‐specific sequences within the 5′ leader region. The oligonucleotide primers were used as followed to generate 188 bp amplicons (Weigel *et al*., 2013): cDNA synthesis (5′‐GTAGGCCTTTACCCCACCAA‐3′), reverse (5′‐GAGCAAGCTCCCTTCATCC‐3′); and forward (5′‐AGCAACGCAAGTAAGACTCG‐3′). All primers were synthesized and purified by Invitrogen, USA. For quantitative real‐time PCR, each 20 μl reaction mixture contained 10 μl 2× PowerUp SYBR Green PCR Master Mix (Applied Biosystems, CA, USA), 0.6 μl of each gene‐specific primer and 2 μl of genomic DNA or cDNA templates that had been added to a total reaction volume of 20 μl. The PCR reaction conditions were performed as followed: 2 min at 50 °C and then 95 °C for 2 min, followed by 40 cycles of 15 s at 95 °C, 15 seconds at 56 °C and 1 min at 72 °C. A melting curve analysis was performed by increasing the temperature from 60 to 95 °C. The standard curve was generated using 10‐fold dilutions of genomic DNA extracted from *P. aeruginosa* PAO1. Threshold cycle (*C*_*t*_) values were analysed automatically with ABI Step‐One‐Plus Real‐Time System software (Version 2.3). To evaluate the effect of PMA and pre‐rRNA under different experimental conditions, the mean cycle threshold differences (Δ*C*_*t*_) and Δ Log were calculated by subtracting *C*_*t*_ values (or log genome copies number) obtained from the PMA‐treated (or stimulated with nutrients) sample from that of the PMA‐untreated (or non‐stimulated with nutrients) sample for each sample pair as follows: Δ*C*_*t*_ (or ΔLog) = w/o – with PMA treatment (or Δ*C*_*t*_ = *C*_*t*_ non‐stimulated – *C*_*t*_ stimulated with nutrients).

### Statistical analysis

Data interpretation was performed using Microsoft Excel 2010 (Microsoft, Redmond, WA). The hypothesis that the means from the two sets of data would be significantly different was tested using Student's t‐test. Results with P values of < 0.05 were considered to be significant. The error bar in this study's figures show standard deviations from two or three independent replicates.

## Conflict of interest

None declared.
